# Cuproptosis-related gene subtypes predict prognosis in patients with head and neck squamous cell carcinoma

**DOI:** 10.1186/s40463-023-00655-4

**Published:** 2023-09-12

**Authors:** Chi Wang, Yu Zhou

**Affiliations:** 1 Department of Oral and Maxillofacial Surgery, School & Hospital of Stomatology, Wenzhou Medical University, Wenzhou, 325000 Zhejiang China; 2 Department of Orthodontics, School & Hospital of Stomatology, Wenzhou Medical University, 373 West College Road, Wenzhou, 325000 Zhejiang China

**Keywords:** Head and neck squamous cell carcinoma, Cuproptosis, Genes, Prognosis, Survival model

## Abstract

**Background:**

Head and neck squamous cell carcinoma (HNSCC) is the sixth most common cancer worldwide. A novel form of copper-dependent and reactive oxygen species (ROS)-dependent cell death, cuproptosis, has been described in many cancers. The roles and potential mechanisms of cuproptosis-related genes (CRGs) are still unclear in HNSCC.

**Method:**

We downloaded TCGA datasets of HNSCC genomic mutations and clinic data from The Cancer Genome Atlas. Based on the Cuproptosis-related differentially expressed genes in HNSCC, we constructed a prognostic signature.

**Results:**

Eight CRGs have been identified as associated with the prognosis of HNSCC. According to Kaplan–Meier analyses, HNSCC with a high Risk Score had a poor prognosis. Furthermore, the AUC of the Risk Score for the 1-, 3-, and 5- year overall survival was respectively, 0.70, 0.71, and 0.68. TCGA data revealed that T cell functions, such as HLA, cytolytic activity, inflammation regulation, co-inhibition, and co-stimulation, differed significantly between members of the low and high groups. The immune checkpoint genes PD-L1, PD-L1, and CTLA-4 were also expressed differently in the two risk groups.

**Conclusions:**

A CRG signature was defined that is associated with the prognosis of patients with HNSCC.

**Graphical abstract:**

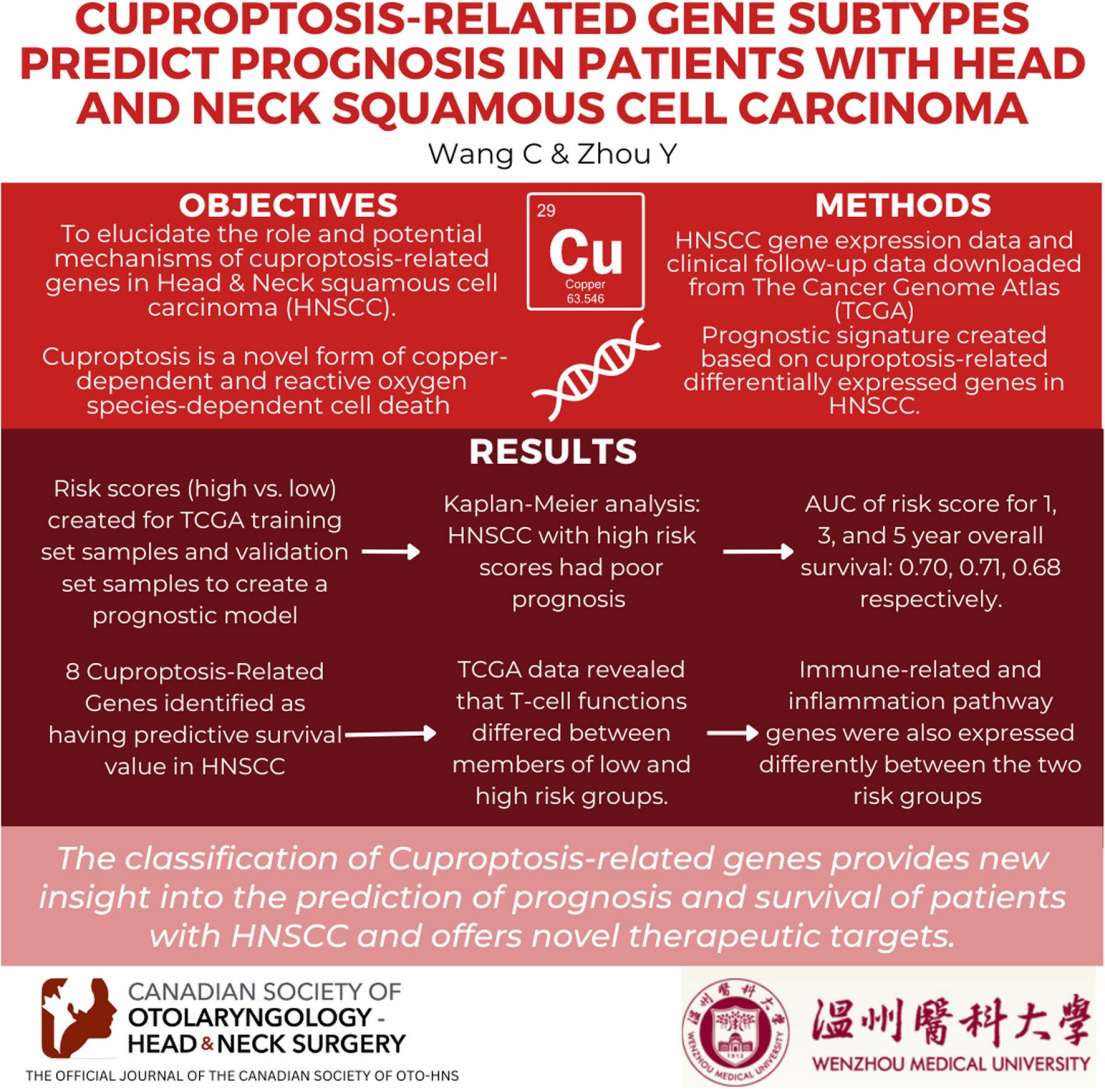

**Supplementary Information:**

The online version contains supplementary material available at 10.1186/s40463-023-00655-4.

## Introduction

Squamous cell carcinoma of the head and neck is the sixth most common cancer worldwide, and the 5-year survival rate is less than 50% despite surgery [1]. Head and neck squamous cell carcinoma (HNSCC) arises from the mucous membranes of the mouth, pharynx, and larynx, primarily due to the consumption of tobacco and alcohol. In HNSCC, clinicopathological characteristics such as extracapsular nodal spread, positive margins, multiple positive nodes, or perineural/vascular invasion have been found to be prognostic factors, most of which lack biological or clinical validation [2]. The lack of accurate biomarkers for early diagnosis or suboptimal preclinical models has limited the effective management of HNSCC. Currently, several antitumor drugs can induce apoptosis of cancer cells. To overcome tumor cell resistance and uncover new and efficient prognostic biomarkers for HNSCC, it is particularly important to investigate other forms of cell death.

Copper has been found to be involved in many important physiological processes, such as mitochondrial respiration, antioxidant defense, and the biosynthesis of hormones, neurotransmitters, pigments, and cell death [3]. A recent study has found that copper can induce another type of cell death that differs from apoptosis, autophagy, or ferroptosis, and has been defined as Cuproptosis, as it involves the tricarboxylic acid cycle [4, 5]. Previous studies have shown that copper participates in three fundamental cancer processes: cell proliferation, angiogenesis, and metastasis [6, 7]. Copper as a cofactor plays an important role in the motility and invasiveness of tumor cells, as well as many angiogenic mediators [3]. Dysregulation of copper metabolism is associated with cancer risk and tumor growth. Cancer cells exhibit copper addiction compared to normal cells, which is the over-reliance on copper for proliferation, which opens a new therapeutic frontier for cancer treatment. Cu ion levels are higher in tumor tissue and serum of cancer patients including oral cancer, bladder, breast, cervical, lung, pancreatic, ovarian, prostate, thyroid, and gastric cancer, than in healthy subjects [8–11]. Other studies have shown that increased copper intake is significant in promoting cancer growth in breast, prostate, pancreatic, colorectal, ovarian, lung, and head and neck cancers [12–16]. Furthermore, severe copper deficiency induced by a low copper diet depresses the immune system of mice, which increases cancer burden. Given that copper preparations are used in the treatment of different types of cancer with a good therapeutic effect, it is crucial to explore biomarkers for HNSCC to better stratify patients and provide personalized treatment. Currently, very few studies have investigated the involvement of Cuproptosis in HNSCC. Advanced research technology, such as microarrays and bioinformatic analysis, has widely been used to screen and identify differentially expressed genes (DEGs) in diseases. We explored DEGs involved in cancer progression in HNSCC.

Since copper-induced death mechanisms are shared by genetic models of copper homeostasis dysregulation, copper homeostasis-related classification may impact on prognosis and the immune response of patients with HNSCC [3]. Therefore, identifying CRG signatures may help to elucidate the causes of heterogeneity in HNSCC. Our study aimed to develop a prognostic marker for HNSCC, which was capable of predicting conventional and immunotherapy prognoses. By screening immunorelated hub genes associated with patient prognosis by weighted gene coexpression network analysis (WGCNA) using HNSCC transcriptome data, we developed an prognostic index based on CRGs, which we defined the immunorelated gene prognostic index (IRGPI).

## Methods

### Data sources and preprocessing

The HNSCC gene expression data and the clinical follow-up data were downloaded from the public database -The Cancer Genome Atlas (TCGA) database. The studies involving human participants were reviewed and approved by ethics committee of Hospital of Stomatology, Wenzhou Medical University(NO.2022010). A total of 514 HNSCC samples with prognostic information were retained after excluding the samples lacking information on survival time and those with survival time less than 30 days. Furthermore, the GSE65858 head and neck cancer dataset from the NCBI GEO database obtained using the GPL10558 Illumina HumanHT-12 V4.0 expression beadchip sequencing platform [17], which contained 270 samples with prognostic information for subsequent validation analysis was retrieved for our analysis.

### Expression differences and correlation analysis of cuproptosis genes

Nineteen CRGs (DBT, ATP7B, FDX1, LIPT1, ATP7A, DLAT, LIPT2, LIAS, DLD, NFE2L2, SLC31A1, PDHB, MTF1, PDHA1, GLS, NLRP3, CDKN2A, GCSH, and DLST) were obtained from the available literature [4]. We observed the differences between these genes in HNSCC versus normal tissues and calculated the Pearson direct correlation coefficient for the 19 genes.

### Identification of cuproptosis subtypes in HNSCC

Tumor subtype analysis was performed on the samples using unsupervised hierarchical clustering Version 1.54.0 [18], for the 19 Cuproptosis genes obtained in Sect. "Expression differences and correlation analysis of Cuproptosis genes" above, to obtain the optimal Cuproptosis subtypes (K value), where the K value range was set from 2 to 6.

### Verification of the cuproptosis score of molecular subtypes (verification of rationality)

For the 19 Cuproptosis genes obtained in 2.2 above, GSVA algorithms [19] (version: 1.36.2) were used to calculate each enrichment fraction of head and neck cancer samples, in order to represent the Cuproptosis rate in each sample. The Wilcoxon test was then used to compare and analyze the differences in the Cuproptosis score among different Cuproptosis subtypes to further verify clinical associations with Cuproptosis subtypes.

### Correlation analysis of prognosis and clinical data of different subtypes

Evaluation of the correlation of survival prognosis of different subtypes between the sample group with the Kaplan–Meier curve method was performed using the R3.6.1 language survival pack Version2.41-1 [20].

### Comparison of the immune microenvironment between different subtypes

The concept of tumor immune microenvironment is that there are a large number of immune cells surrounding and with the tumor tissue. Several relationships exist between these immune cells and tumor cells. There are many types of immune cells; thus, the so-called immune microenvironment, or an analysis of immune infiltration, essentially determines the proportion of immune cells in the tumor tissue. In this analysis, the following two algorithms were used to evaluate the status of the immune microenvironment in HNSCC samples, and differences in infiltration between different subtypes were tested using the Wilcoxon test.

We calculated the composition of 22 types of immune cells in HNSCC using CIBERSOR [21]. Based on linear support vector regression, CIBERSORT allows deconvolution of the expression matrix of immune cell subtypes. The ESTIMATE and the Estimator [22] algorithm tool are used to calculate the stromal score and immune score of tumor samples based on expression data of immune cell types.

### Comparison of immune checkpoint genes and HLA family gene differences between subtypes

Immune checkpoint genes (PD1(PDCD1), PD-L1(CD274); CTLA-4(CTLA4); CD278(ICOS); TIM3(HAVCR2); LAG3; CD47; BTLA; TIGIT; MYD1(SIRPA); OX40(TNFRSF4); 4-1BB(TNFRSF9); B7-h4 (VTCN1)) and HLA family genes were extracted from HNSCC expression data;). The Wilcoxon test was used to compare the differences ins expression of immune checkpoint genes and HLA family genes among subtypes.

### Identification of specific genes between subtypes

To observe the different molecular mechanisms that may exist between different Cuproptosis subtypes, we used the linear regression and empirical Bayes method, provided by the limma package (Version 3.10.3) [23] to perform differential analysis of gene expression between different subtypes, and the corresponding P-value and log2fold change (FC) of the gene were obtained. In addition, the Benjamini–Hochberg method is used for multiple test correction, and the corrected P-value was corrected via Kolmogorov–Smirnov test, expressed as adjusted *P*-value. Fold change and significant difference were determined and statistical significance was set at *P* < 0.05 and |log_2_FC|> 1.The differentially expressed genes (DEGs) were defined as those specifically expressed between subtypes.

### Functional enrichment analysis

For DEGs obtained in step “Identification of specific genes between subtypes”, we performed a Gene Ontology (GO) function analysis and Kyoto Encyclopedia of Genes and Genomes (KEGG) pathway enrichment analysis with ‘clusterProfiler’ of the R package (version 4.0.5) [24] to explore the entry of functional pathways involved in key genes. The Gene Ontology (GO) is an internationally standardized classification of gene functions that generates a dynamically updated Controlled Vocabulary for describing organism products. Genes are described by three ontologies in GO: molecular function, cellular component, and biological process. With a *P*-value < 0.05 and FC > 2 as thresholds, the most significant TOP10 genes were selected for our analysis.

### Identification of prognostic genes

Based on the DEGs identified from in Step “Identification of specific genes between subtypes”, a Univariate Cox regression analysis was performed in the Survival Package v2.41–1 to select specific genes whose expression was significantly correlated with the survival prognosis for subsequent analysis.

### Construction and performance verification of the prognostic model (Risk Score)

By applying cross-validation based on prognosis-related genes in Step Identification of prognostic genes, key genes were selected using LASSO analysis (lars package v1.2) with penalty tuning parameters (lambda value). Furthermore, a Risk Score was established by multivariate stepwise Cox regression analysis with the survminer package (Version 0.4.9).

$${\text{Risk Score }} = {\text{ h}}0\left( {\text{t}} \right) \, *{\text{ exp }}\left( {\beta {\text{1X1 }} + \, \beta {\text{2X2 }} + \, \cdots \, + \beta {\text{nXn}}} \right)$$

In this formula, β refers to the regression coefficient, ho(t) is the benchmark risk function; H (t,X) is the risk function related to X(covariable) at time t.

This formula was used to calculate the Risk Scores for the training set samples (TCGA) and the validation set samples. Using the Risk Score formula, we divided each sample into low- and high-risk groups. Kaplan–Meier analysis was used to compare the survival rates of high- and low-risk patients.

### Independent prognostic analysis

By integrating clinical data with HNSCC, the correlation between the risk groups and clinical information (age, sex, stage, ImmuneScore,Clinical_N, Clinical_T,grade) was analyzed. Univariate and multivariate independent prognostic analyses were performed to determine the relationship between clinical features and the risk groups. The Forest plot of the R package was used to visualize the results of independent prognostic factor analysis with *P*-values < 0.05.

### The construction of the nomogram model

A nomogram was created using the R software package and the nomogram function of the rms library v5.1-2 [25] based on the independent prognostic factors selected in Step “Independent prognostic analysis”. Nomograms are used to assess the correlation between independent survival factors and prognosis and survival prediction.

### Analysis of drug sensitivity

The cancer drug sensitivity genomic database was used to estimate the sensitivity of each patient to chemotherapy drugs. The half maximum inhibitory concentration (IC50) was calculated using the drc in the R package [26]. IC50 differences of 138 chemotherapeutic drugs were compared between different risk groups with the Wilcoxon test.

### Prediction of the efficacy of immunotherapy

The response to checkpoint blockade immunotherapy of each patient was predicted using the Tumor Immune Dysfunction and Exclusion (TIDE) tool, defined as TIDE prediction scores, and the Wilcoxon test was used to compare TIDE scores in different risk groups.

### Gene set enrichment analysis

Gene Set Enrichment Analysis (GSEA) is a calculation that is used to assess whether differences between biological states can be statistically significant [27]. In this study, GSEA was used to analyze the significant enrichment of Hallmark gene sets (H) (v7.4.symbols) in the entire genome between the high- and low-risk groups, |NES|> 1 and FDR < 0.05 were considered statistically significant.

### Associations between risk groups and cuproptosis subtypes

The Sankey diagram was drawn by combining the subtype grouping (group) and the high- and low-risk group (risk group) of the samples using the 'ggalluvial' package [28] to observe the relationship between the cuproptosis subtype and the HNSCC high-risk group.

## Results

### Expression patterns of the cuproptosis genes

**Fig. 1:**
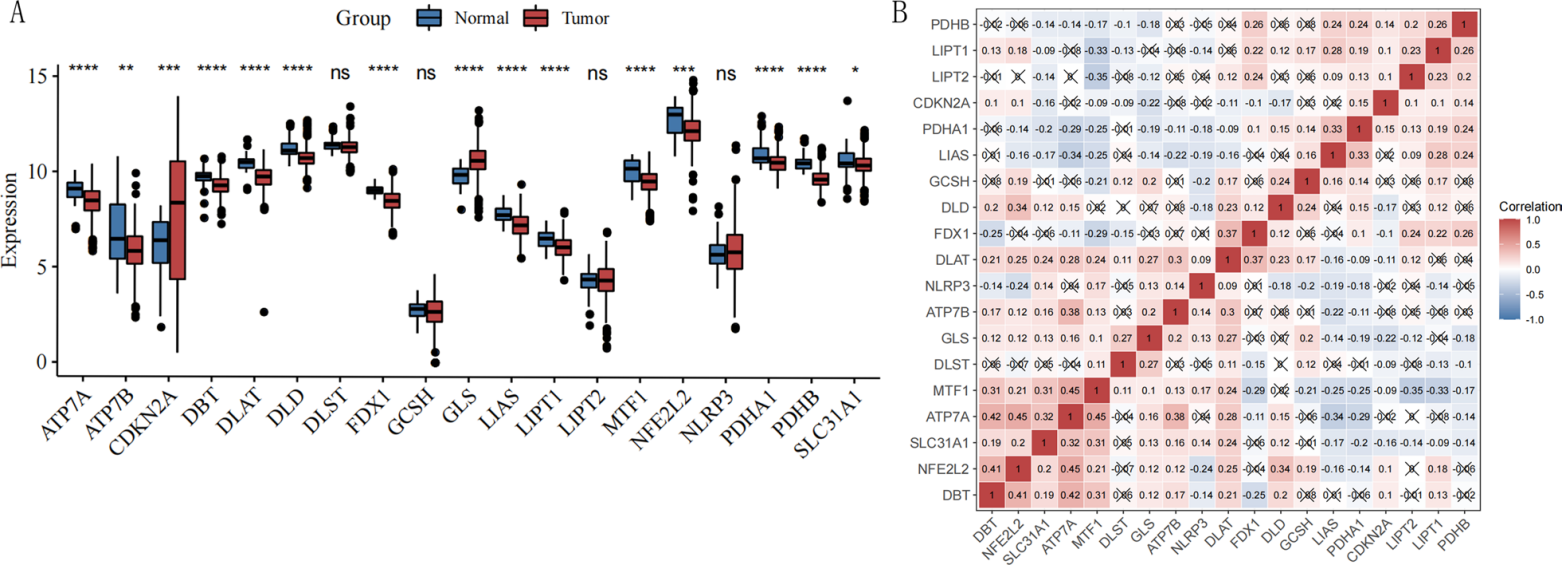
**A** Boxplot illustrating 19 Cuprotosis-related genes expression in HNSCC samples and associated normal controls. * indicates a *p* value of less than 0.05; ** indicates a *p* value of less than 0.01. **B** The heatmaps showing the expression levels of the 19 Cuprotosis-related genes. X stands for no biological significance

We identified a set of 19 putative CRGs (with different expression patterns. As shown in Fig. 1A, the expression of 15 (GLS, FDX1, LIPT1, ATP7B, SLC31A1, LIAS, DLAT, PDHA1, NFE2L2, PDHB, DLD, MTF1, CDKN2A, ATP7A, and DBT) of the 19 genes were significantly different between HNSCC and normal samples (*P* < 0.05). Of these, 2 genes (CDKN2A, GLS) were up-regulated, and the other 13 genes were down-regulated in HNSCC samples. To explore the association between different genes associated with cuproptosis, we describe correlation patterns between the 19 genes (Fig. 1B). The information is shown in Additional file 1: Appendix 1.

### Identification of cuproptosis subtypes in HNSCC

**Fig. 2:**
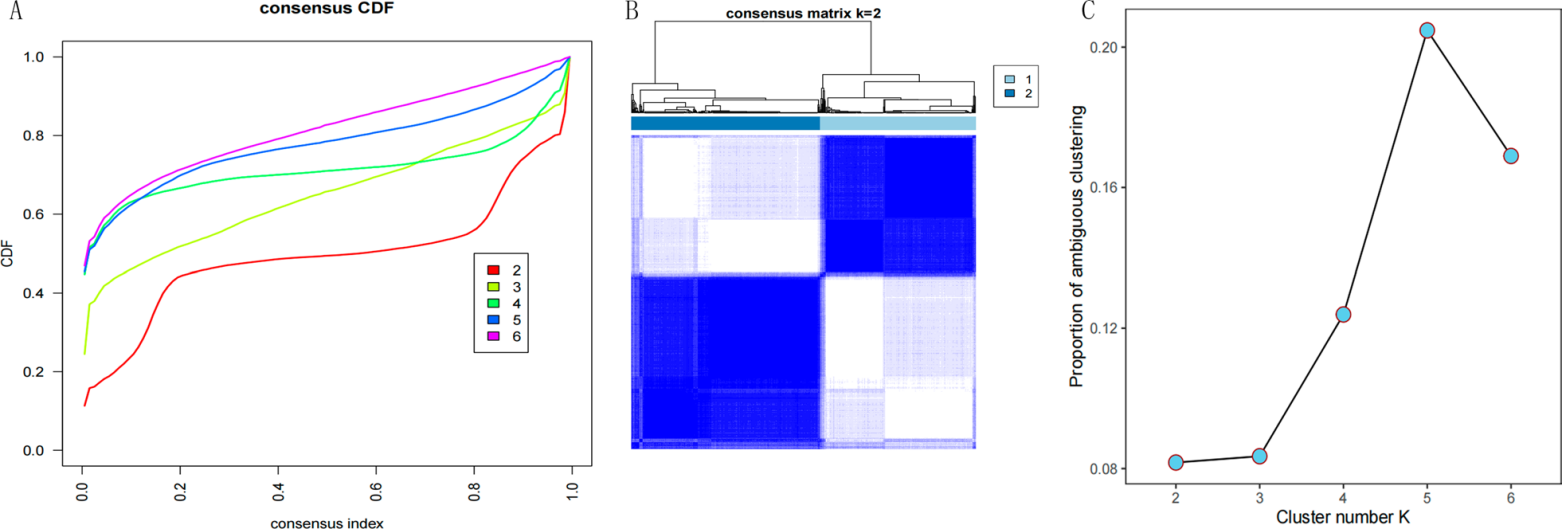
**A** Consensus clustering cumulative distribution function (CDF) for k = 2–6. **B** Consensus clustering matrix for k = 2. **C** PAC validation curve

For the 19 CRGs identified in Step Expression patterns of the cuproptosis genes, an unsupervised cluster analysis was performed on HNSCC samples (Fig. 2A), setting the K value range from 2 to 6, the optimal K = 2 is selected. As shown in Fig. 2B, two different subtypes (C1 and C2) were obtained and contained 232 and 282 HNSCC samples, respectively (Additional file 1: Appendix 2). Proportion of ambiguous clustering (PAC) verification curve as shown in Fig. 2C further verifies the stability of the clustering results. The minimum value of PAC, namely with an optimal K, is 2.

### Comparison of cuproptosis scores

**Fig. 3:**
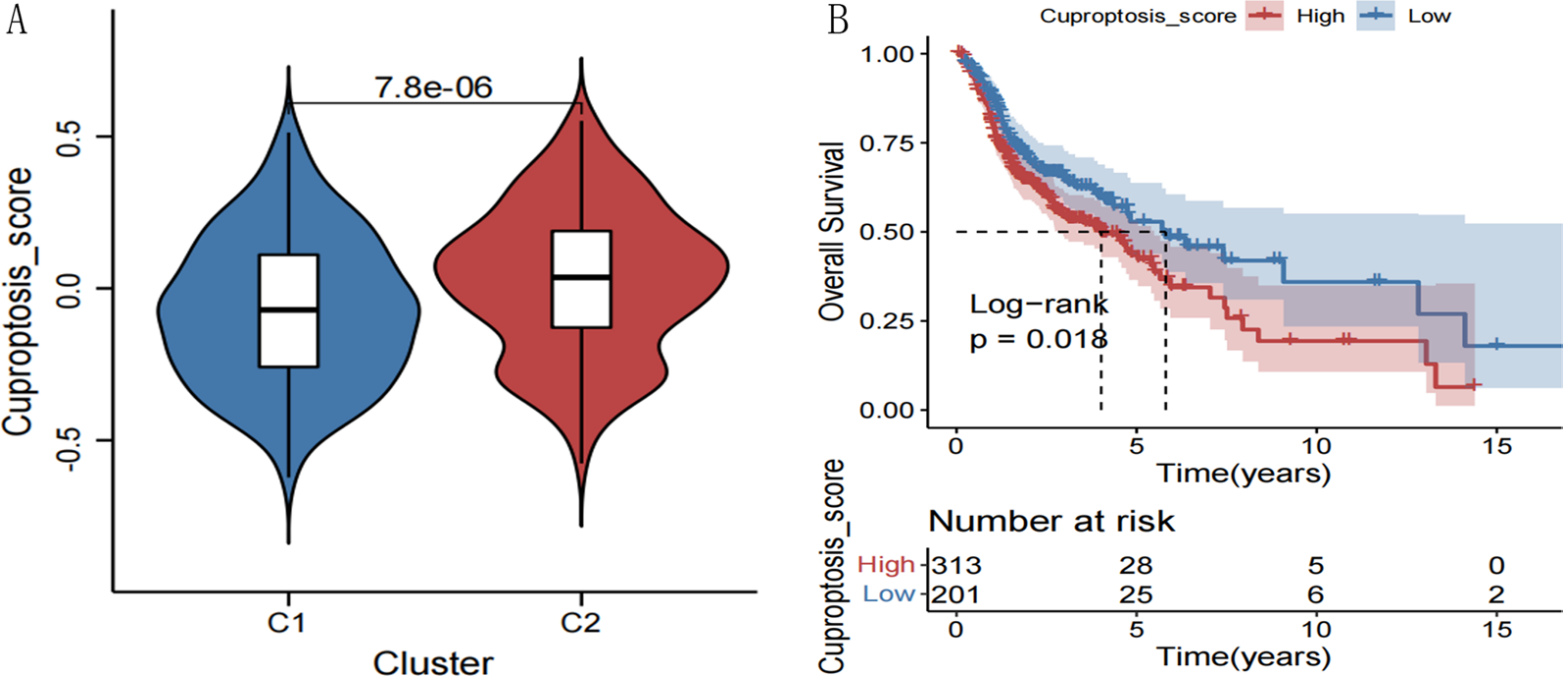
**A** Comparison of cuproptosis scores between different subtypes. **B** KM survival curves for cuproptosis scores

For the 19 cuproptosis genes, the GSVA algorithm was used to calculate the enrichment score in each HNSCC sample to represent the cuproptosis score for each sample, as shown in Figure 3. The cuproptosis score was significantly different between the two subtypes (Additional file 1: Appendix 3), with the cuproptosis score lower in cluster C1. Therefore, the stratification of the cuproptosis subtypes was further confirmed.

### Survival analysis and clinical correlation of subtypes

**Fig. 4:**
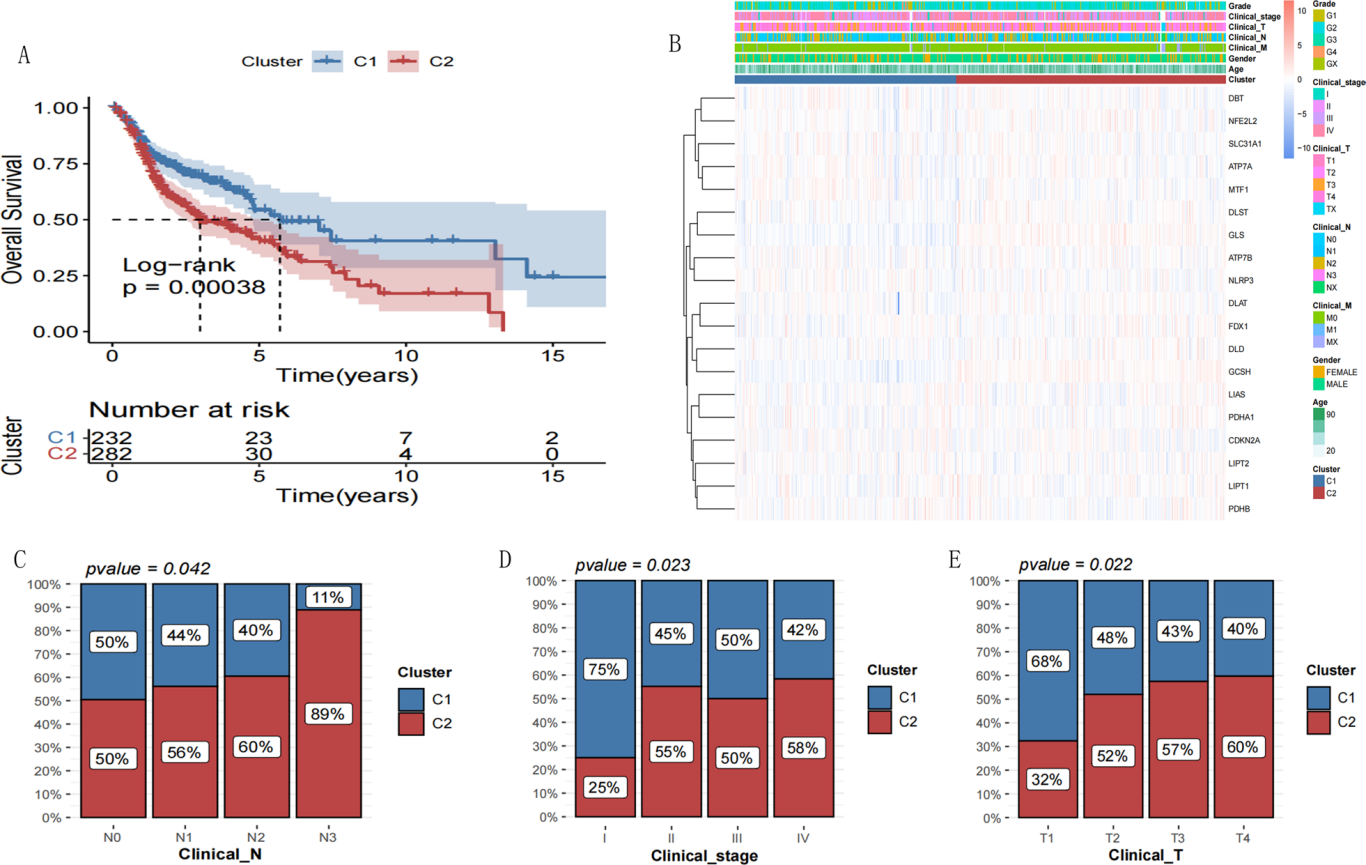
**A** KM survival curves of different subtypes. **B** Heat map of the 50 most differentially expressed genes. Blue represents low expression; red represents high expression. **C** The clinical features between the two subtypes of patients

Kaplan-Meier curve analysis was used to assess the correlation between survival and prognosis of different subtypes (Figure 4A). The survival prognosis information for different subtypes was significantly different, and cluster C2 was associated with a poor prognosis. The distribution of the expression of the 19 cuproptosis genes evaluated in each subtype was plotted on a heatmap (Figure 4B). For all HNSCC samples, clinical information of the samples was classified and the correlation analysis between the subtypes and clinical characteristics of the samples was performed (Additional file 1: Appendix 4). As shown by the Chi-square test results in Figure 4C, there were significant differences in the TMN staging (Clinical_N, Clinical_T, and Clinical_stage) between the two subtypes.

### Comparison of the immune microenvironment between cuproptosis subtypes

**Fig. 5:**
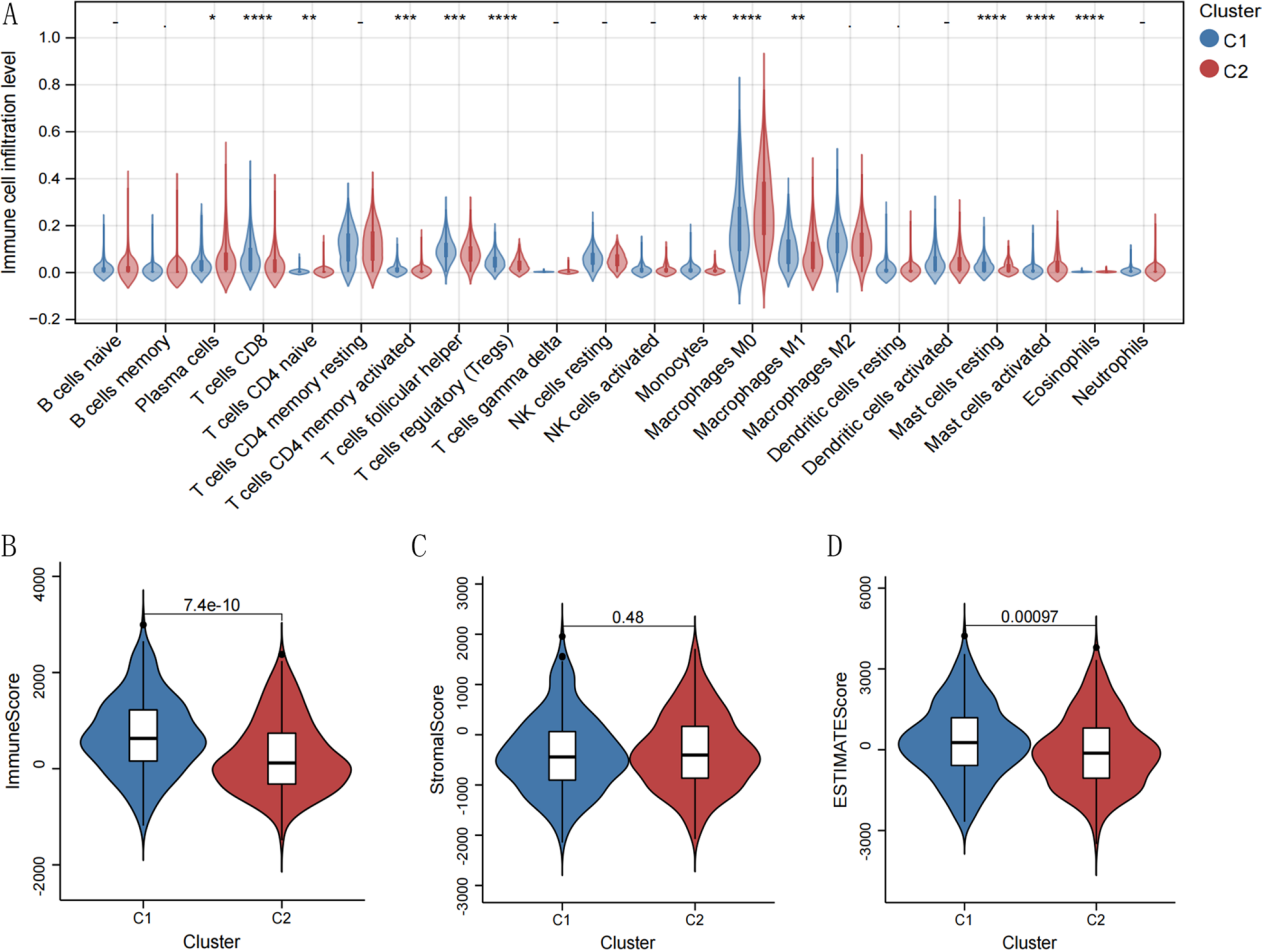
**A** Immune cell types with significant differences between the two subtypes using CIBERSORT. **B** The violin plots of different infiltration levels of immune cells with immune scores and stromal scores in different subtypes.* indicates a *p* value of less than 0.05; ** indicates a *p* value of less than 0.01;*** *P* value of less than 0.001

Based on the expression profile data of the HNSCC samples, the CIBERSORT algorithm was used to calculate the immune cell types of each sample and the proportion of each of the 22 immune cell types was obtained. The information is shown in Additional file 1: Appendix 5. Differences in the proportion of various immune cells were compared between different subtypes. Based on *P* < 0.05 as the threshold, 12 distinct immune cell types (DICs) were identified, and Fig. 5A shows the comparison results between groups. Using the Estimate algorithm, immune and matrix scores were calculated, as shown in Additional file 1: Appendix 6. The differences in immune and stromal scores between different subtypes were then analyzed, as shown in Fig. 5B. Cluster C1 was found to have higher percentages of CD8+ T cells, follicular helper T cells, T-regs, monocytes, and mast cells resting, and lower percentages of activated mast cells, macrophages, CD4+ T cells, and plasma cells than cluster C2.

### Analysis of immune checkpoints and HLA family genes between subtypes

**Fig. 6:**
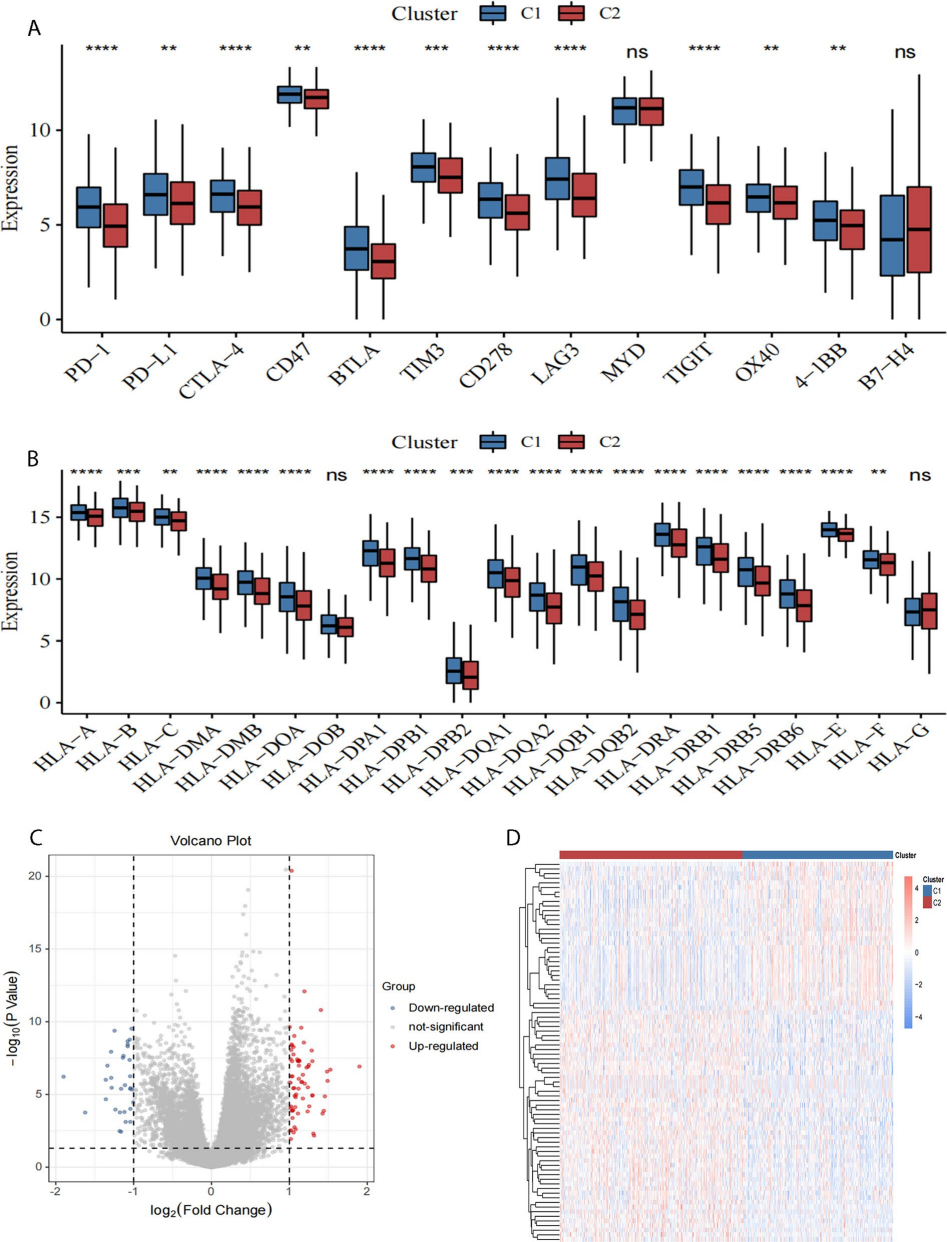
**A** Differential expression of the immune checkpoint genes in different subtypes. **B** The expression difference of HLA family between different subtypes. * indicates a *p* value of less than 0.05; ** indicates a *p* value of less than 0.01; *** *P* value of less than 0.001. **C** Volcano plot for differential gene expression.Blue represents low expression; red represents high expression. **D** Heat map of differential gene expression

The expression of immune checkpoint genes extracted from TCGA dataset is shown in Additional file 1: Appendix 7. Differences in immune checkpoint gene expression between different subtypes were compared, with *P* < 0.05 as the threshold and a total of 11 immune checkpoint genes (CD278, LAG3, BTLA, PD-1, 4-1BB, CTLA-4, CD47, TIM3, PD-L1, TIGIT, and OX40) with significant differences were obtained; the comparative analysis between subtypes is shown in Fig. 6A. The expression of the HLA family gene was compared between different subtypes using the Wilcoxon test. The comparisons between subtypes are shown in Fig. 6B and the list is provided in Additional file 1: Appendix 8.

The expression of 19 HLA family genes (HLA-A, HLA-B, HLA-C, HLA-DMA, HLA-DMB, HLA-DOA, HLA-DPA1, HLA-DPB1, HLA-DPB2, HLA-DQA1, HLA-DQA2, HLA-DQB1, HLA-DQB2, HLA-DRA, HLA-DRB1, HLA-DRB5, HLA-DRB6, HLA-E, and HLA-F) was significantly different between clusters C1 and C2, and all genes were upregulated in cluster C1.

### Identification of differentially expressed genes between subtypes

Using the method described in Step “Comparison of immune checkpoint genes and HLA family gene differences between subtypes”, a differential gene analysis was performed between the two subtypes (Fig. 6C, Additional file 1: Appendix 9), and 101 differentially expressed genes were obtained. Among these, the expression of 69 genes such as GCSH, B4GALNT1 was up-regulated and that of 32 genes such as PTPRCAP, ZNF683 was down-regulated.

### Enrichment analysis of functional pathways

**Fig. 7:**
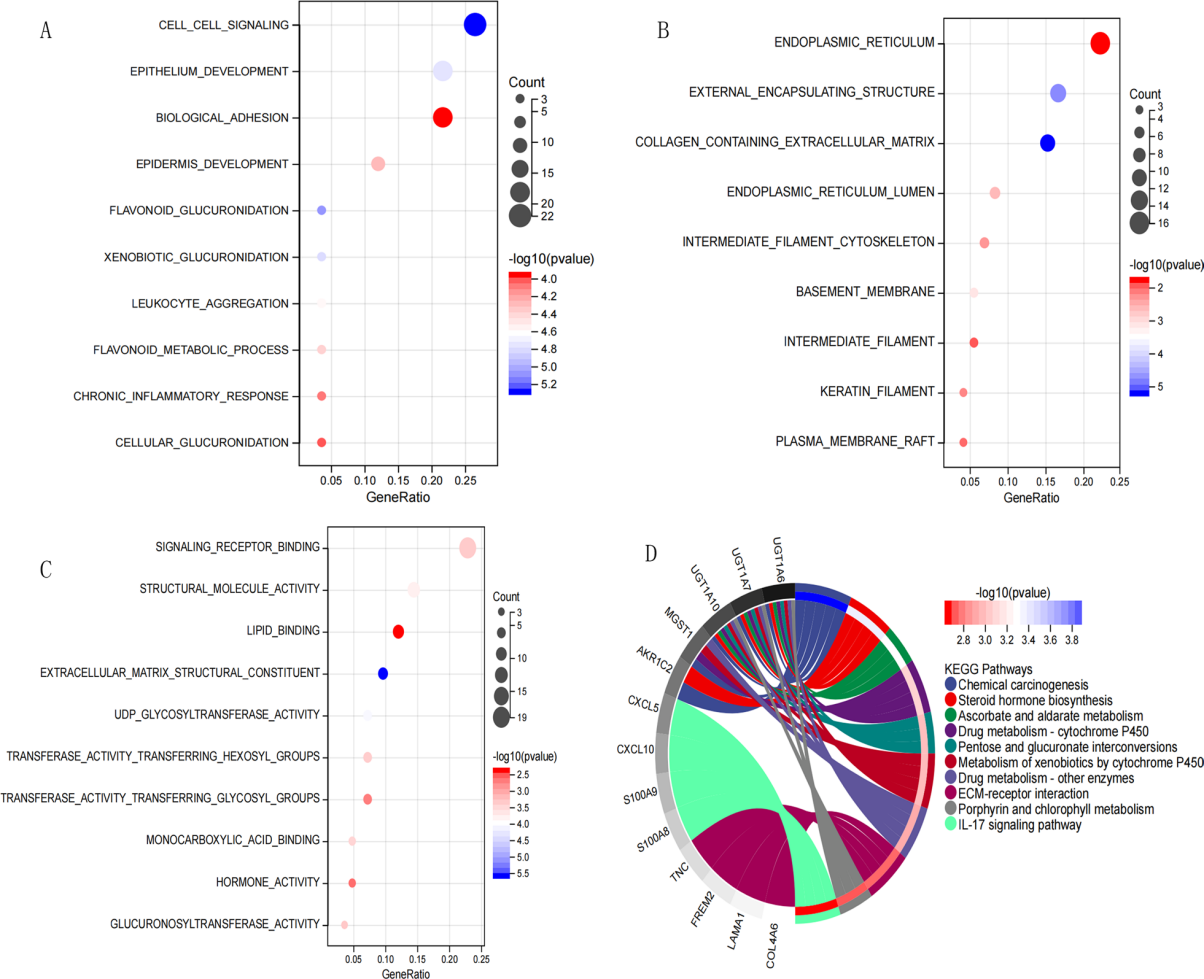
GO functional and KEGG pathway enrichment of differentially expressed genes. **A**–**C** Bubble chart, the horizontal axis represents the proportion of those genes, the vertical axis represents the pathway name, the size of the dot indicates the number of genes expressed in the pathway, and the color of the dot corresponds to the different Qvalue range. **D** Chord diagram, the outer circle shows differentially expressed genes involved in the pathway

GO function and KEGG signal pathway enrichment analyses were performed for the DEGs obtained in Step “Identification of differentially expressed genes between subtypes” to explore the functional terms involving the key genes. Enrichment results are shown in Fig. 7, which lists the Top10 results having a *P*-value < 0.05 and FC > 2 as thresholds. A total of 146 significantly correlated DEGs enriched in 9 cell components, 26 biological processes involved in 15 KEGG signaling pathways, and 17 molecular functions were selected. The information is shown in Additional file 1: Appendix 10. Among the biological processes, the DEGs were mainly enriched in cell–cell signaling, epithelium development, and biological adhesion. Regarding the cellular components, the DEGs were significantly enriched in the collagen-containing extracellular matrix and the external encapsulating structure. For molecular functions, DEGs were significantly enriched in signaling receptor binding. Figure 7 shows all the detailed results of the GO term enrichment analysis. In addition, 5 DEGs were significantly enriched in chemical carcinogenesis pathways.

### Identification of genes significantly associated with prognosis

**Fig. 8:**
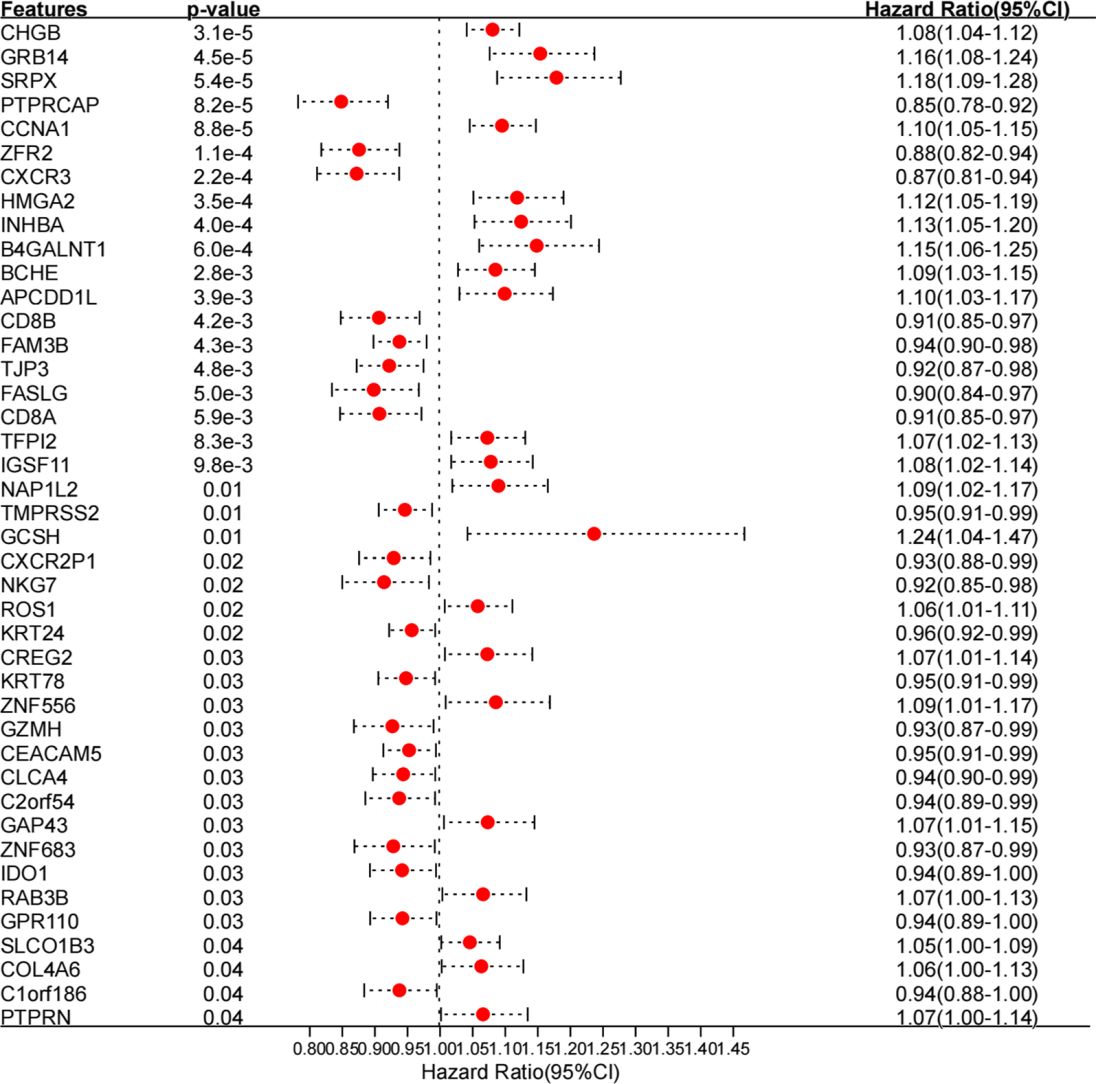
Univariate regression forest plot of the 42 prognostic genes

Among the DEGs identified in Sect. "Identification of differentially expressed genes between subtypes" above, a total of 42 genes, including CHGB, GRB14, PTPRCAP, with significant prognostic correlation were selected by univariate Cox regression analysis using the R3.6.1 language survival package (v2.41-1) with *P* < 0.05 as the threshold (Fig. 8), and the gene list is provided in Additional file 1: Appendix 11.

### Construction of the prognostic model and performance verification

**Fig. 9:**
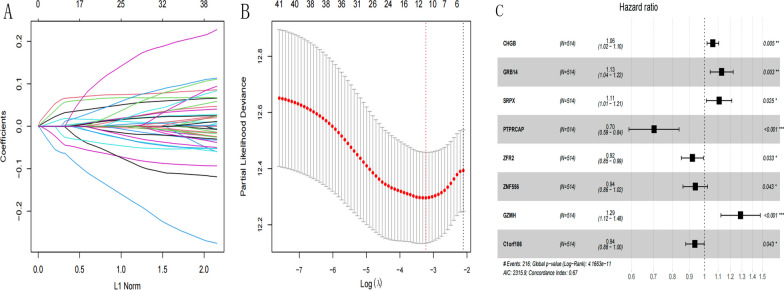
**A** LASSO coefficient profiles of the 42 prognostic genes. **B** Partial likelihood deviance for the LASSO coefficient profiles. Two vertical dotted lines indicate lambda. min(the red line on the left) and lambda.1se (The black line on the right). **C** Forest plot of multivariate Cox regression analysis of 8 model genes

**Fig. 10:**
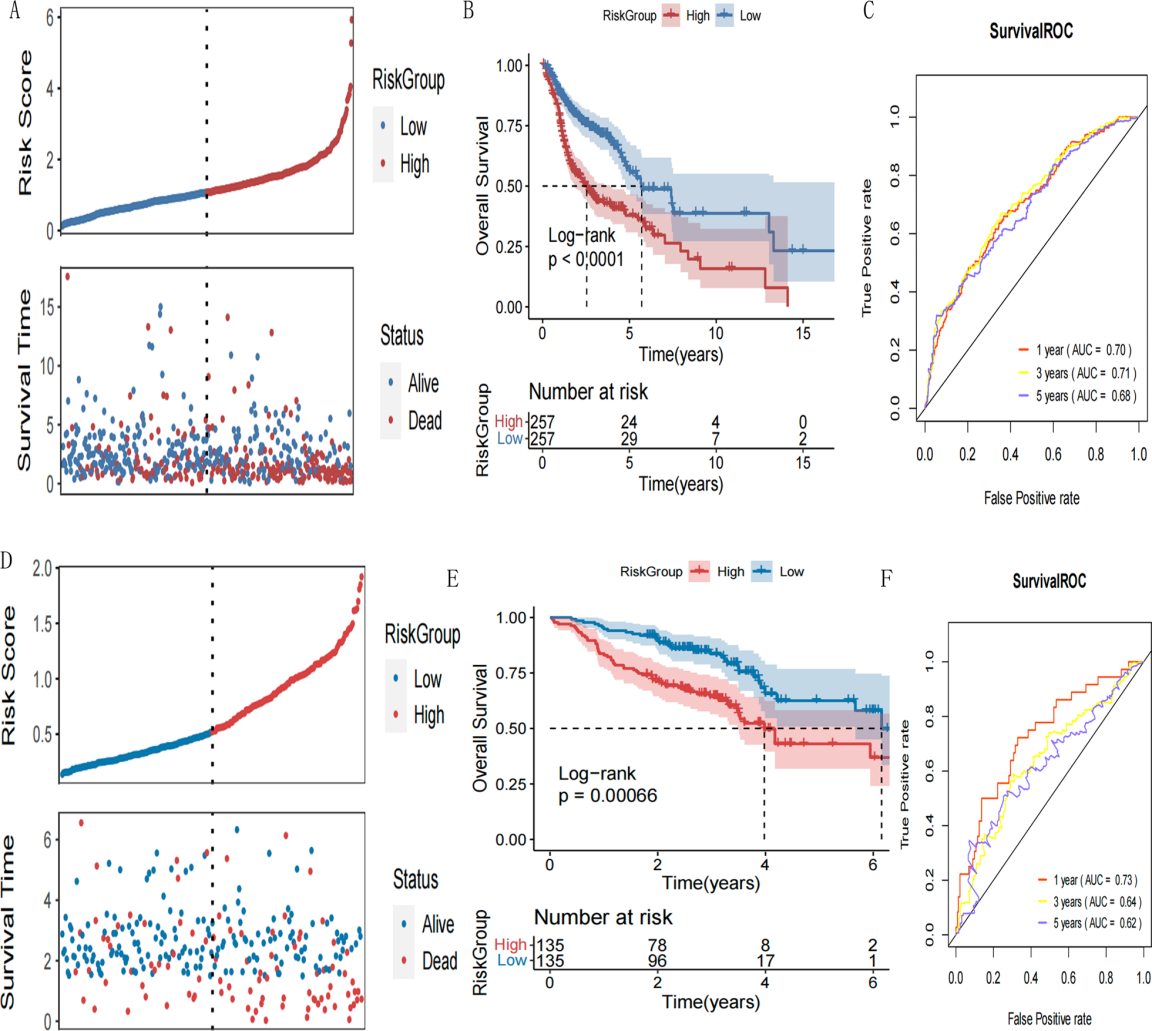
**A** The risk score distribution, and patients survival status in TCGA training set. **B** KM survival curves based on riskscore prediction model. **C** ROC curves of the training set for 1-year, 3-year and 5-year survival. **D** The risk score distribution, and patients survival status in GEO validation set. **E** KM survival curves based on riskscore prediction model. **F** ROC curves of the validation set for 1-year, 3-year and 5-year survival

**Fig. 11:**
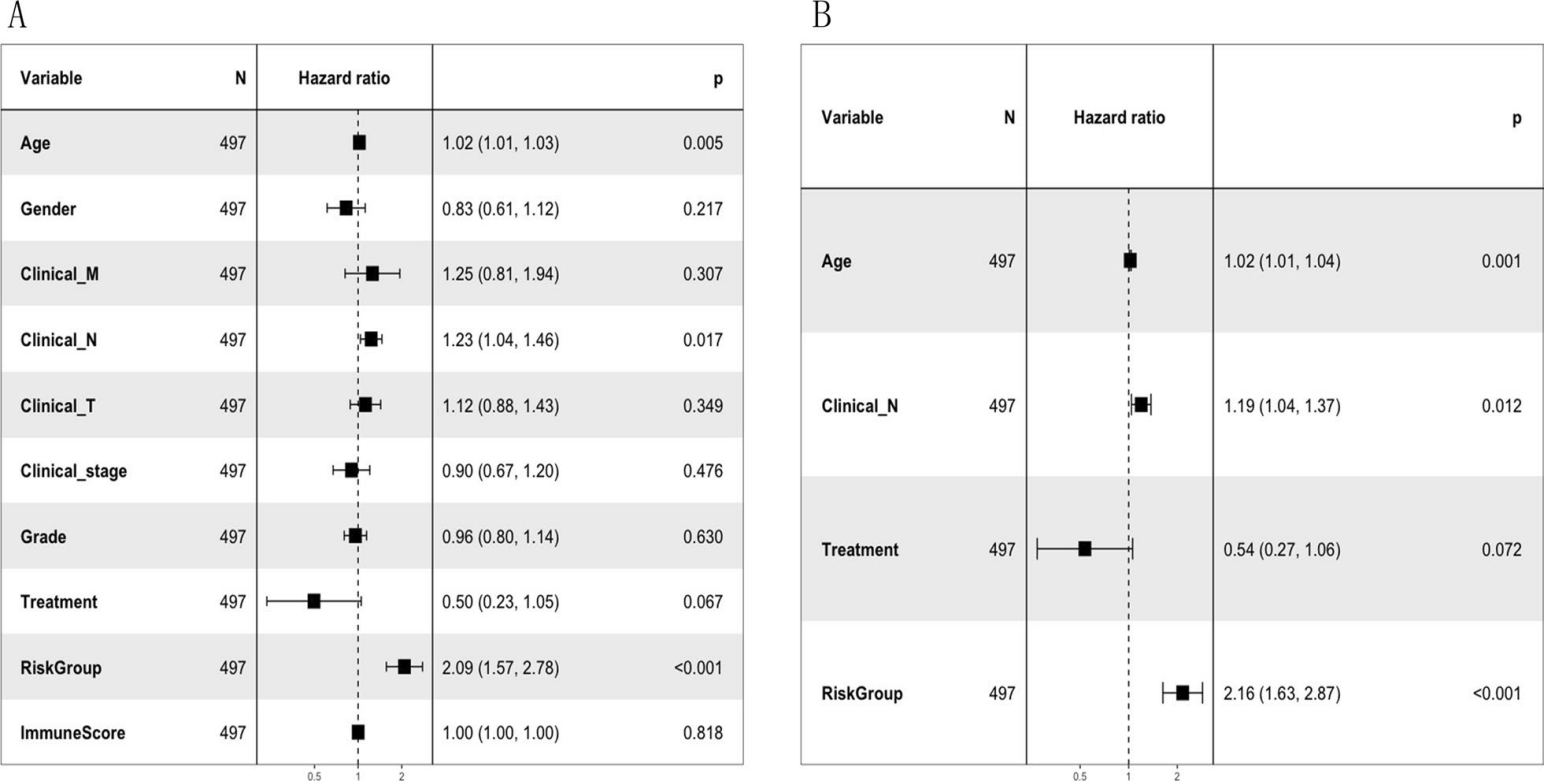
Forest plots of univariate (**A**) and multivariate cox regression analysis (**B**) of clinical information

Based on the expression data of 42 prognostic genes identified in 3.9 above, the LASSO algorithm was used to select optimized genes (Figs. 9) and 11 key genes were obtained. Stepwise COX regression algorithm was used to select the optimal gene combinations (Fig. 9). Finally, 8 model genes (CHGB, GRB14, SRPX, PTPRCAP, ZFR2, ZNF556, GZMH, and C1orf186) were obtained. The Risk Score model was then constructed based on the regression coefficients of these 8 genes and their expression levels in TCGA training data set. Of these, CHGB, GRB14, SRPX, and CCNA1 were positively correlated with the Risk Score. The Risk Score of each patient was calculated and the samples in TCGA training set and the GEO verification set were divided into High-risk (Risk Score higher than the median value of the Risk Score) and Low-risk (Risk Score equal to less than the median value of the Risk Score) groups. The distribution of the Risk Score value and the survival time distribution for each group is shown in Fig. 10A and D. The Kaplan–Meier curves were then used to assess the association between the High- and Low-risk groups and the actual prognoses of the patients. The Kaplan–Meier curves for each data set are shown in Fig. 10B and E, respectively. ROC curves of 1-, 3-, and 5 year survival based on genetic prognostic characteristics are shown in Fig. 10C and F. In TCGA training set and the GEO validation set, the different risk groups based on the prediction of the Risk Score model were significantly correlated with the actual prognosis; the high-risk group had a worse prognosis. The scores and grouping of the Risk Score in the training and validation sets are shown in Additional file 1: Appendix 12 and 13, respectively.

### Prognostic independence analysis

The clinical information of all TCGA head and neck cancer samples was extracted (Additional file 1: Appendix 14). Univariate Cox regression analysis was performed on the clinical factors and for each risk group using the R3.6.1 language survival package. Factors with *P* < 0.05 were selected for multivariate Cox regression to identify significant independent prognostic factors (Fig. 11). Multivariate Cox regression analysis confirmed that the risk group was an independent prognostic factor after adjustment for other clinicopathological factors.

### Development of a nomogram to predict survival based on independent prognostic factors

**Fig. 12:**
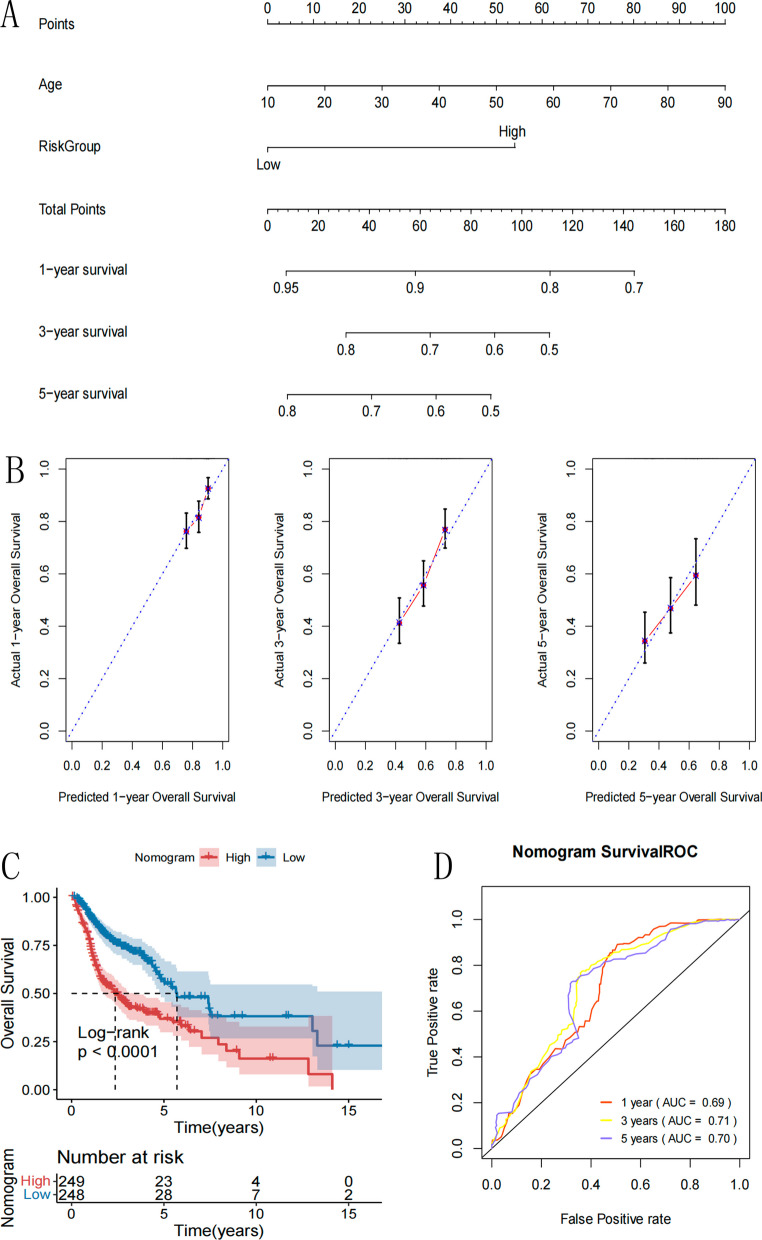
**A** The nomogram to predict overall survival based on independent prognostic factors. **B** Calibration plot for nomogram predicted and actual survival rate. Vertical axis represents actual survival, and horizontal axis represents the nomogram-predicted survival. **C** KM survival curves based on nomogram prediction model. **D** ROC curve of nomogram model predicting1-year, 3-year and 5-year survival

To further analyze the correlation between age and risk group, which were significantly correlated with prognosis and survival prognosis, age and risk group were included in the construction of a Nomogram survival model, as shown in Fig. 12A. Integrating various clinical indicators into the "Total points" axis in the first row predicted the survival of the samples. The consistency between the 1-, 3-, and 5-year survival rates predicted by the survival model and the actual 1-, 3-, and 5-year survival rates was analyzed and verified (Fig. 12B). Figure 12C shows that the nomogram is significantly associated with the patient's prognosis. The 1-, 3-, and 5-year ROC curves of the nomogram of the nomogram are shown in Fig. 12D.

### Analysis of drug sensitivity

**Fig. 13:**
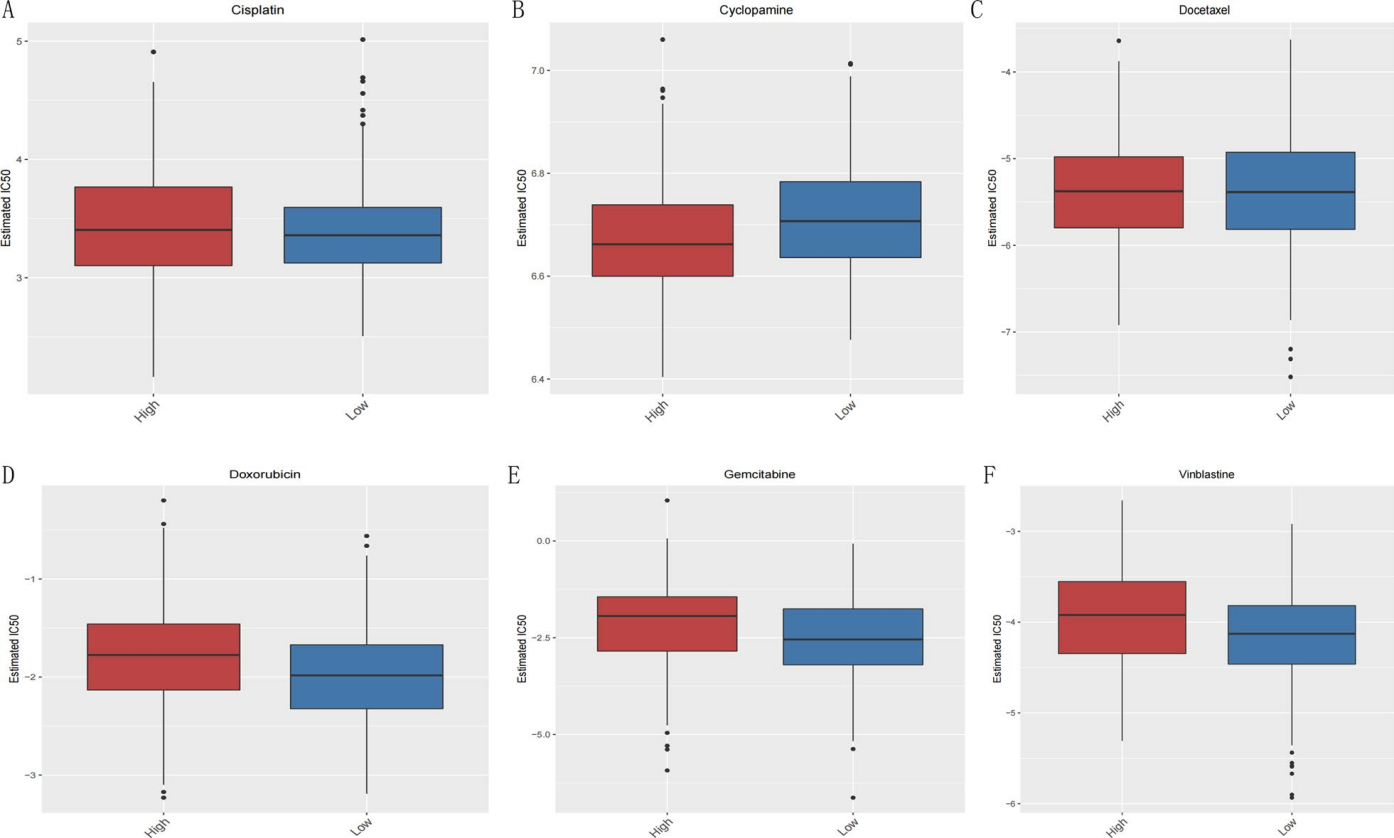
IC50 values of six chemotherapeutic drugs in different risk groups

The sensitivity of each patient to chemotherapeutic agents was estimated based on the cancer drug sensitivity genomics database, and the IC50 was quantified using the pRRophetic package in R. We compared the differences in the IC50 levels of 138 chemotherapeutic agents (Additional file 1: Appendix 15) and the six common chemotherapeutic agents identified are shown in Fig. 13.

### Prediction of the efficacy of immunotherapy

**Fig. 14:**
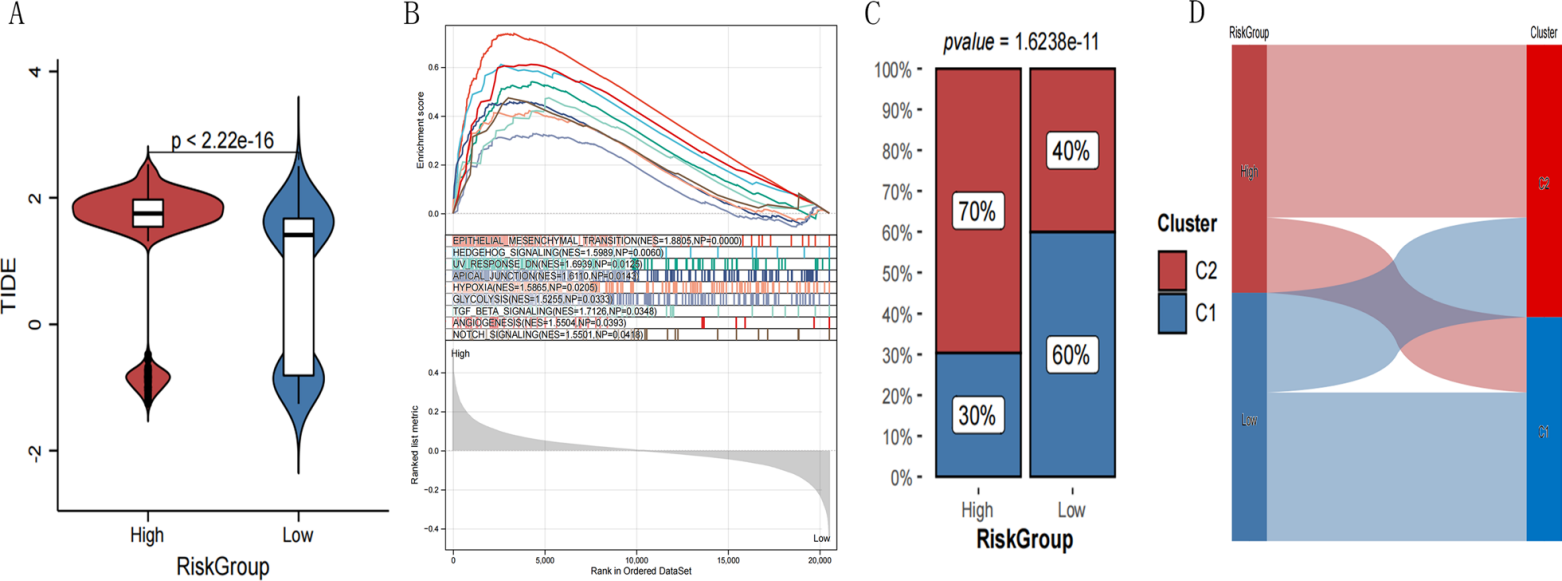
**A** Comparison of the TIDE score in different risk groups. **B** Hallmark gene sets with a significant enrichment. **C** Distribution between Cluster and RiskGroup

Using the TIDE algorithm, a TIDE score was calculated for each patient with HNSCC to predict his response to immune checkpoint therapy (Additional file 1: Appendix 16). As shown in Fig. 14A, subjects in the high-risk group showed higher TIDE scores than those in the low-risk group.

### GSEA enrichment analysis

GSEA analysis revealed that there were 9 sets of hallmark genes with significant differences in the different risk groups with a value of *P* < 0.05 and the normal enrichment score > 1 as thresholds (Fig. 14B and Additional file 1: Appendix 17). The gene sets of the high-risk group samples were enriched in the epithelial-mesenchymal transition and the Hedgehog signaling pathways.

### Association between high- and low-risk groups and cuproptosis subtypes

The relationship between cuproptosis subtype (Cluster) grouping and high and low risk groups (risk group) was evaluated (Fig. 14C). Through a one-to-one correspondence of the samples, the Sankey diagram was drawn as shown in Fig. 14D and in Additional file 1: Appendix 18. The high-risk groups had a significantly higher proportion of genes belonging to the C2 subtype.

## Discussion

In this comprehensive study, DEGs involved in cuproptosis were found to have predictive survival value in HNSCC and indicate potential response to immunotherapy. According to our study, the expression of cuproptosis genes was significantly lower in most tumors and may have a prognostic value in HNSCC. In addition, the expression of CRGs was closely associated with immune and inflammation-related pathways, immune cell infiltration, and several immune-related genes. Before cuproptosis was discovered,Eric Winquist’s research [29] showed that, Other than cetuximab, no targeted agents and radiosensitizers studied in RCTs were shown effective for HNSCC.But in this research,we found that there was an association between cuproptosis and sensitivity to cancer chemotherapy drugs. Thus, our study shows that cuproptosis might be a biomarker of prognosis and may predict response to immunotherapy.

As cancer therapies develop, clinicians now have the option to treat patients with HNSCC by targeting immune checkpoints. Many anticancer drugs (Dabrafenib, vemurafenib,Val-boroPro) show a profound effect on programmed cell death, implied that programmed cell death,such as apoptosis,autophagy,ferroptosis,may play a role in cancer therapy [30]. Previous studies have shown that we can reduce cisplatin resistance in cancer cells, and enhance the sensitivity of tumor cells to chemotherapeutic drugs by promoting ferroptosis in tumor cells [31]. With the development of anticancer therapies that selectively induce cancer cell death, cuproptosis may become a new target for the treatment of HNSCC. In this study, our unsupervised cluster analysis subdivided HNSCC into two groups C1, C2, which differ significantly in cuproptosis gene expression. Clinical_N, Clinical_T, and Clinical_stage showed significant differences between these two subtypes. Combined TCGA and GEO data sets demonstrated better prognoses and associated with the C1 cluster. In addition, we constructed a prognostic model with eight genes associated with prognosis using a LASSO Cox regression analysis. Based on the prognostic model, the patients were divided into two risk groups, and those with a lower Risk Score had better clinical characteristics and greater immune cell infiltration. Additionally, in our study, we found that age, sex, lymph node metastasis, and risk group are important prognostic factors in HNSCC. Furthermore, we established a nomogram survival model to predict the survival of patients with HNSCC based on independent prognostic factors. The high-risk group was found to have poorer clinical outcomes. Furthermore, we found that the high-risk group had a significantly higher proportion of genes present in the C2 subtype. A better understanding of the molecular mechanism of HNSCC and an accurate prognosis stratification can be achieved based on the findings of this study. This could lead to novel strategies using immunotherapy for patients with HNSCC. By comprehensively evaluating CRGs and clinical characteristics, we not only developed an accurate prognosis model, but we also provide a rationale for immunotherapy as a treatment method.

Cuproptosis is a new type of cell death that has the potential of providing a new approach to the treatment of cancer. Recent studies have found that these cuprotosis-associated genes may be associated with the development of diverse tumors, including bladder cancer, hepatocellular carcinoma, breast cancer, lung cancer, colorectal cancer and so on [32–36]. However, several key issues, such as the interconnection between cuproptosis and host immunogenicity and other cell deaths, remain unresolved by current research. A deeper understanding of its regulatory system and its specific mechanisms for controlling different types of cancer is needed. Thus, this study explored whether a CRG signature could predict the prognosis of HNSCC patients. To date, there have been a few reports suggesting that FDX1 is an important regulator of cuproptosis [4, 5], which is consistent with our finding that FDX1 is significantly decreased in HNSCC. FDX1 expression has been shown to be highly correlated with lipoylated proteins, while protein lipoylation contributes to copper-induced cell death. In our study, a significantly decreased expression of FDX1 was observed in HNSCC patients.So as another important regulator of cuproptosis-LISA.

Cuproptosis subtypes established on the basis of Cuproptosis scores showed significantly different prognoses. Glycine cleavage system H protein (GCSH), an important protein complex associated with the tricarboxylic acid cycle, which is highly associated with Cuproptosis, showed higher expression in the C1 subtype [4, 5]. Currently, there are very few studies on the correlation between GCSH and cancer, and only one article has described the correlation between GCSH and breast cancer [37]. The study reported that the level of GCSH expression in breast cancer tissue was higher than that in normal breast tissue, which was similar to our results. These findings imply that GCSH may play an important role in cancer development.

There were significant differences in immune scores between these two subgroups. Previous studies have shown that the immune microenvironment of the tumor is closely related to the prognosis of patients with HNSCC. The immune microenvironment plays an important role in the development, prognosis, and immunotherapy of tumors. Several changes in the immune response have been observed in patients with HNSCC, suggesting that it is an immunosuppressive cancer [38, 39]. Infiltrating immune cells can influence cancer aggressiveness [40]. Our study showed that there is no difference in the level of infiltration of naïve B cells and B cell memory cells between the two clusters, while the level of infiltration of CD8 T cells, follicular helper T cells, Tregs, resting NK cells, and activated NK cells of the C1 subtype is much higher than that of the C2 subtype. These results implied that cell immunity plays an important role in the antitumor immune response, and high expression of CD8 T cells and NK cells is associated with a good prognosis, which remains consistent with previous findings [41–43].

Eleven of 13 immune checkpoint genes were up-regulated and most HLA genes showed higher expression in the C1 subtype, which appeared to have a better prognosis in patients with HNSCC. Elements of the tumor microenvironment disrupt CD8-mediated immunity, which contributes to tumor immune escape [44] and peripheral blood of patients with HNSCC showed suppressive activity of regulatory T cells [45]. Interestingly, our study found that immunostimulator, CD8 T cells, PD-L1, and immunosuppressive factors, PD-1, CTLA-4 and Tregs were highly expressed in cluster 1, which may indicate that the C1 subtype was characteristic of an active immune response. Furthermore, in our prognostic model, high-risk patients mainly possessed subtype C2, while low-risk patients possessed subtype C1. The high-risk group also had higher TIDE values, meaning that they were prone to immune evasion, leading to a worse prognosis. The result implied that immunotherapy targeting the tumor microenvironment is feasible.However, whether there is a direct relationship between cuproptosis and tumor immune microenvironment needs to be further studied.

A tight regulation of copper homeostasis ensures that sufficient amounts are available for cuproprotein biosynthesis, and that oxidative stress is limited. Currently, copper ionophores, such as disulfiram (DSF) and elesclomol, are being used to treat cancer and have been shown to be effective [46, 47]. Researchers have shown that DSF or DSF/Cu2+ results in increased intracellular ROS accumulation, which further supports its use as an HNSCC treatment regimen [48]. Further research showed that these anticancer drugs work by selectively transporting Cu ions to the mitochondria and increasing their local ROS levels [49], sharing a similar cuproptosis mechanism described above, which is consistent with our findings that cuproptosis may have a positive effect in the treatment of HNSCC. When new copper-targeting cancer drugs are developed, it will be crucial to determine which biomarker of copper homeostasis represents the most reliable therapeutic target.

Through functional pathway enrichment analysis, we found that differential DEGs were primarily enriched in cell–cell signaling, epithelium development, and biological adhesion, indicating that these genes could act as oncogenes, promoting HNSCC invasion. As for cellular components, the DEGs were significantly enriched in the collagen-containing extracellular matrix and the external encapsulating structure, which was consistent with the biological functions mentioned above. For molecular functions, differential DEGs were significantly enriched in signaling receptor binding, which participate in cell-to-cell signaling.

Although our study provides important insights for evaluating cuproptosis and the prognosis of patients with HNSCC. There are still some limitations that will need to be addressed in future work. First, the small sample size decreased the power of the statistical analysis. We are continuing to recruit patients and anticipate that a larger sample size would confirm our present results. Second, we need further functional studies to understand the exact regulatory mechanism of cuproptosis in HNSCC. The association identified in this research requires validation by further studies combined with people and animal experiments. There is no complete clinical information available, such as HPV/tobacco, treatment, histological prognostic models, so their model is biased by definition to exclude other prognostic factors. Similarly, no protein level information can be obtained.

Overall, this study offers new insights into the relationship between the cuproptosis and the prognosis of patients with HNSCC. Its findings could contribute to a better understanding of the mechanism of cuproptosis in HNSCC.The discovery of the mechanism of cuproptosis provides a direction for future drug research, and cuproptosis related drugs, that can induce the cuproptosis may have some application prospects in the future treatment of cancers.

## Conclusions

The classification of genes related to Cuproptosis provides new insight on the prediction of prognosis and survival of patients with HNSCC and offers novel predictive and therapeutic strategies for these patients.

### Supplementary Information

**Additional file 1.** The original data of this study.

## Abbreviations

HNSCC: Head and neck squamous cell carcinoma
ROS: Reactive oxygen species
CRGs: Cuproptosis-related genes
DEGs: Differentially expressed genes
WGCNA: Weighted gene coexpression network analysis
IRGPI: Immunorelated gene prognostic index
TCGA: The Cancer Genome Atlas
FC: Fold change
GO: Gene ontology
KEGG: Kyoto Encyclopedia of Genes and Genomes
IC50: Half maximum inhibitory concentration
TIDE: Tumor Immune Dysfunction and Exclusion
GSEA: Gene set enrichment analysis
PAC: Proportion of ambiguous clustering
GCSH: Glycine cleavage system H protein
